# Water quality assessment of aquatic ecosystems using ecological criteria – case study in Bulgaria

**DOI:** 10.1080/13102818.2014.974383

**Published:** 2014-11-04

**Authors:** Sonya Damyanova, Iliana Ivanova, Nadka Ignatova

**Affiliations:** ^a^ Department of Plant Pathology and Chemistry, Faculty of Ecology and Landscape Architecture, University of Forestry, Sofia, Bulgaria; ^b^ Department of Microbiology, Faculty of Biology, Sofia University “St Kliment Ohridski”, Sofia, Bulgaria

**Keywords:** aquatic ecosystem, critical loads, *Pseudomonas putida* growth inhibition test, heavy metals, risk assessment

## Abstract

Four aquatic ecosystems (two rivers and two dams) situated in the western part of Bulgaria were investigated over a three years’ period. The River Egulya and Petrohan dam are situated in mountainous regions at about 1000 m altitude, and are not influenced by any anthropogenic sources. Petrohan dam is a site for long-term ecosystem research as a part of Bulgarian long-term ecological research network. The other two systems belong to populated industrial areas. The River Martinovska flows through a region with former long-term mining activity, while Ogosta dam is near a battery production factory. Both the geochemical and geographical ecosystems’ conditions are different, and their social usage as well. Ogosta dam water is used for irrigation and Petrohan dam for electric supply. The ecosystem sensitivity to heavy metals was evaluated by a critical load approach. Two criteria were used for risk assessment: critical load exceedance and microbial toxicity test. All studied ecosystems were more sensitive to cadmium than to lead deposition. The potential risk of Cd damage is higher for Petrohan dam and the River Egulya, where critical load exceedance was calculated for two years. *Pseudomonas putida* growth inhibition test detected a lack of toxicity for all studied ecosystems at the time of investigation with the exception of the low water September sample of the River Martinovska. The fast bacterial test is very suitable for a regular measurement of water toxicity because of its simplicity, lack of sophisticated equipment and clear results.

## Introduction

The quantity of fresh water in the world is decreasing every year with the global climate changes. The quality of surface water is very important for people who use it as a source to meet their everyday needs in the household and agriculture. Critical concentrations of heavy metals in surface water are dangerous for living organisms. Some elements, such as Cu, Mn, and Zn, are part of enzyme systems, and they are not toxic in low concentrations. Other metals, like Pb, Cd and Hg, are extremely toxic because they are not common and recognizable by organisms, and only some micro-organisms have metabolic paths for their removal.

Heavy metal compounds are usually present in very small concentrations in nature. Mining activity and industrial development over the last decades have caused adverse effects on the environment and human health. Heavy metal pollution has been detected everywhere: in the atmosphere, hydrosphere and lithosphere, causing harm to living organisms. Toxic metals adsorbed on fine particulate matter could reach far distances and may affect isolated locations. Mountain ecosystems are very sensitive because of the lack of defensive mechanisms against the anthropogenic impact.

The critical load approach is used by the coordination centre for effect of the International Cooperative Programme (ICP) modelling and mapping to access the harmful effect of heavy metals and protect the ecosystems.[1,2] This approach has been successfully used to solve problems with acidity and eutrophication in Europe in the context of the convention on Long-Range Transboundary Air Pollution (LRTAP).[3] According to Nilsson and Grennfelt,[4] the critical loads are defined as ‘the highest pollutant load in the long term that does not cause a significant harmful effect on a specific element or ecosystem function at the present knowledge’. They are derived from critical limits using an effect-based approach. For aquatic ecosystems, appropriate assessment is toxicological, based on total metal concentration in fresh water for protecting all living organisms (algae, crustaceans, worms, fish and top predators). A simple steady-state mass balance model is used for the calculation of critical loads. It is based on the balance of all relevant input and output metal fluxes of the studied ecosystem. This balance means no further accumulation of metals or ecosystem damages in the long term.

Critical loads show the regional distribution of ecosystem sensitivity to heavy metal pollution. To assess the impact of heavy metals on a specific receptor, it is necessary to compare their deposition rates to critical loads. Exceedances of critical loads indicate ecosystems at risk of damage. When the atmospheric depositions are higher than the critical loads, an ecosystem receives a larger amount of metals than it does under conditions of normal functioning. Exceedance of critical loads shows a potential risk for living organisms in the future, because accumulated metals will exceed their critical limit and cause harmful effects. The lack of exceedance means no damage at that moment.

Chemical and physical analyses do not meet the needs for fast and cheap assessment of general water quality, as they require sophisticated equipment and preliminary information about possible pollution. Biotests with micro-organisms could show the overall water quality in a cheap and fast experiment even if there is no information about pollution or no preliminary investigations. Microbial tests do not need special equipment and could reliably tell the status of a system. That is why they are often used in quality water assessment. In our investigation, the growth inhibition test with *Pseudomonas putida* was used.[5]

## Materials and methods

### Site description

**Figure 1.**
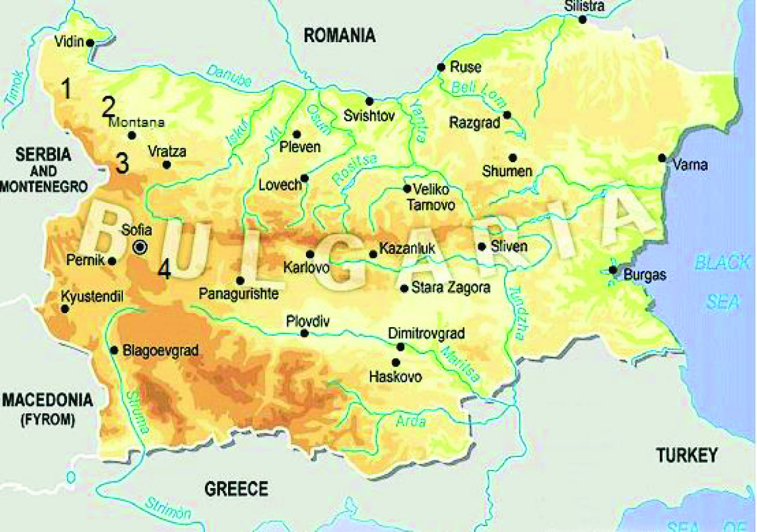
Site location. (1) The River Martinovska, (2) Ogosta dam, (3) Petrohan dam and (4) the River Egulya. Note: Used with permission from Industial Zones Bulgaria, http://www.industrial-zones.com.

The study was carried out at four aquatic catchments situated in the north-western part of Bulgaria, for a period of three years (Figure 1).

Petrohan dam is part of an electric supply system. It is located in an extensively forested region at an altitude of 1480 m, where there are no industrial or agricultural sources of heavy metal pollutants. The dam area is 116 ha. The forest consists mainly of beech and oak species and an insignificant number of Scots pines and spruces. Ogosta dam has 2360 ha of water area and is used for irrigation and industrial supply. It is located at an altitude of 190 m, a few kilometres away from the town of Montana, where there is a battery production factory. The forest consists of mixed stands of beech, oak, hornbeam and a negligible part of Scots pines. The River Egulya is situated at 1200 m altitude. The forest consists mainly of deciduous beech forest and a very small part of spruce. The River Martinovska is located at 485 m altitude in mainly coniferous (70% Scots pine) and beech forests. There has been mining activity there from the medieval ages to the end of the 1990s.

### Sampling, analysis and database collection

Water and river sediment samplings were collected every two weeks for the four aquatic ecosystems in the period 2006–2008. This means at least 20 samples for each water body per year were gathered. For heavy metal determination, each water sample (0.500 dm^3^) was preserved by adding 2 cm^3^ of concentrated HNO_3_ acid, transported in a polypropylene bottle and kept at 4 ºC till analysis. For the measurement of pH, toxicity, suspended particulate matter and river and dam sediments, no preserved samples were used. Bulk precipitations were established using permanently opened polyethylene plastic collectors. Each collector had a collection area of 314 cm^2^ and stood approximately 1.5 m above ground level. To analyse dissolved metal forms, all water samples were filtrated through a 0.45 μm cellulose filter. Water chemistry of the samples was determined as follows: Pb^2+^ and Cd^2+^ ions by using inductively coupled plasma emission spectrometry (ICP-AES); suspended particulate matter, gravimetrically; and pH by means of a pH-meter. Surface water and precipitation samples were initially concentrated 50 times, and were then diluted with concentrated HNO_3_ acid to a volume of 0.010 dm^3^. River and dam sediments were dried at room temperature, sieved through 0.25 mm and 2-g samples were wet digested using a mixture of concentrated HCl and HNO_3_ acids.

### Test micro-organism

*Pseudomonas putida* DSM 50026 is a Gram-negative motile aerobe from the family *Pseudomonadaceae*. It is rod shaped and has a diameter of 0.7–1.1 μm, a length of 2.0–4.0 μm. It is common in surface water and soils, and its optimal temperature for growth is between 23 and 30 °C. The test strain was chosen for its sensitivity to heavy metals and pesticides among 20 natural isolates from the same species. It was adopted as European standard for water quality assessment in 1995.[5]

A water quality test was conducted with inoculums at the exponential phase of strain *P. putida* DSM 50026. The optical density of three different dilutions of the investigated water samples was compared to the control bacterial growth in synthetic medium prepared with distilled water. The effect of investigated river water was determined through measuring the optical density of an exponential bacterial culture at 610 nm (during 16 h ± 1 h).

The tests were conducted with fresh bacterial culture adapted to simple synthetic medium during three pre-cultivations after rich maintaining medium, as described in the standard procedure. The growth inhibition test used mineral nutrient medium with the following components: 2.0 g dm^−3^ of glucose, 500 mg dm^−3^ of NaNO_3_, 120 mg dm^−3^ of K_2_HPO_4_, 60 mg dm^−3^ of KH_2_PO_4_, 20 mg dm^−3^ of MgSO_4_ 7H_2_O and 0.5 mg dm^−3^ of iron citrate. The concentrated solutions of all components were prepared, and, in all tests, equal quantities of each were added, so that the only variable was the quantity of tested water. The inoculum was 10% of the final volume with an optical density of 0.005 at λ = 610 nm. The tested water was added to the final volume of 100 cm^3^, equal in the three replicates. Each water sample was tested in three variants: natural, two- and four-times dissolved. Samples were incubated in a shaker at 180 r/min for 16 h at 25 °С. Optical density was measured at the same wave length against nutrient medium without bacteria (blank sample) as a control. Toxicity was determined in May high water (freshet) and September low water.

An indirect quantification method and a direct diffusion method were also used to determine the quality of tested water.

*Indirect method for bacterial growth inhibition: Koch's method*. The quantity of micro-organisms was determined on sterile nutrient medium at the end of the experiment (16th h). Consecutive decimal dilutions were made and samples were inoculated in nutrient-rich medium. The colony-forming units (CFU) were determined after 24th h cultivation at room temperature.

*Disk-diffusion method*. Fifty microlitres of tested natural and diluted water were dropped on agar plates inoculated with the test micro-organism *P. putida*. Bacterial growth inhibition was detected after 24 h at 23 ºC ± 2 ºC incubation if sterile zones were formed. The method was used to test insoluble water pollutants.

### Calculation of critical load and its exceedance

The method to calculate critical loads of heavy metals is based on the simple steady-state mass balance approach of all relevant input and output metal fluxes of the studied ecosystem.[3,4] The model implies that the critical load equals the net uptake by forest growth plus an acceptable metal outflow water rate, according to the following equation:(1) 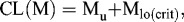
where CL(M) is the critical load of a heavy metal, (g ha^−1^ yr^−1^); M_u_ is the metal net uptake in harvestable parts of plants under critical load conditions, (g ha^−1^ yr^−1^) and M_lo(crit)_ is the critical metal outflow from the catchment, (g ha^−1^ yr^−1^).

Metal net uptake (M_u_) by biomass was calculated by multiplying the annual yield (*Y*_ha_ (kg ha^−1^ yr^−1^)) by the metal content of the harvestable parts of the trees ((M)_ha_, (g kg^−1^ dw)) as follows:(2) 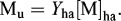



Local research of the metal content in Bulgarian forests shows concentrations of Pb of 0.007 g kg^−1^ for coniferous and 0.006 g kg^−1^ for deciduous species. The relevant values for Cd content are 0.0004 g kg^−1^ and 0.0003 g kg^−1^.

Critical metal outflow from the catchment (M_lo(crit)_, (g ha^−1^ yr^−1^)) was calculated according to the following equation:(3) 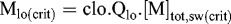



where Q_lo_ is the outflow flux of water from the catchment (m yr^−1^); [M]_tot,sw(crit)_ is the critical limit for the total concentration of heavy metal in the surface water; and clo = 10, is a factor for appropriate conversion of flux units from (mg m^−2^ yr^−1^) to (g ha^−1^ yr^−1^).(4) 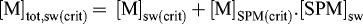
where [M]_sw(crit)_ is the critical dissolved concentration of a heavy metal in surface water (mg m^−3^); [M]_SPM(crit)_ critical total content of a heavy metal in suspended particles (mg kg^−1^); and [SPM]_sw_ is the concentration of suspended particles in surface water (kg m^−3^).

In the calculation process, the values used were 0.38 mg m^−3^ for Cd [6,7] and 11 mg m^−3^ for Pb [8] as critical dissolved concentrations. Net retention in the dams was not taken into account because of the lack of data.[9] It was accepted that the critical total content of a heavy metal in suspended particles equals the sediment concentration of this metal.

The exceedance of the critical loads was calculated as the difference between metal deposition and critical load value.(5) 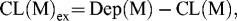



where Dep(M) is the deposition metal rate for the whole year (g ha^−1^ yr^−1^),(6) 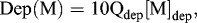
where Q_dep_ is the precipitation amount (mm) and [M]_dep_ is the mean metal concentration (mg dm^−3^).

## Results and discussion

Surface water chemistry showed neutral activity: the two rivers and Petrohan dam had pH values between 6.71 and 6.90 and Ogosta dam, 7.22, respectively. At these pH values, insignificant concentrations of heavy metals were available. The River Martinovska and Petrohan dam, which are mountain water bodies, contained a very small amount of suspended particles (0.0038 g dm^−3^ for the river and 0.0047 g dm^−3^ for the dam). More twice as high was the amount of suspended particles in the River Egulya (0.0101 g dm^−3^) and Petrohan dam (0.0135 g dm^−3^).

The mean calculated critical loads for Pb and Cd for the whole investigated period using steady-state mass balance method are shown in Table 1.

**Table 1.** Mean critical load values for Pb and Cd for the period 2006–2008.

Water ecosystem	Petrohan dam	Ogosta dam	The River Martinovska	The River Egulya
CL(Pb) (g ha^−1^ yr^−1^)	23.75	18.22	23.73	16.37
CL(Cd) (g ha^−1^ yr^−1^)	1.02	0.82	1.14	0.78

As already mentioned, critical loads indicate the sensitivity of ecosystems to anthropogenic impact. The calculated critical loads for Pb (Table 1) were much higher (more than 20 times) than those for Cd. This indicates that all ecosystems are significantly more sensitive to cadmium deposition compared to lead deposition. The tolerance of Petrohan dam and the River Martinovska to Pb input was similar, and at the same time, higher than that of the other studied ecosystems. Therefore, the former two ecosystems could be expected to be able to withstand a potential increase of metal pollution in the near future and survive without damages. The most sensitive amongst all investigated ecosystems was the River Egulya because of its lowest critical load values. The probability of metal accumulation in the future is very high, which increases the risk of adverse effects on the most sensitive water organisms.

The ecosystem tolerance to Cd deposition was not the same as that to Pb tolerance (Table 1). For example, the Martinovska ecosystem had the highest critical load value for Cd among all investigated ecosystems, which was not the same for Pb. The reason is that each metal has specific behaviour depending on the water chemistry. This demonstrates the advantage of the critical load approach because it takes into account all individual features of ecosystem components and functions. In respect of Cd, critical loads were comparable for Ogosta dam and the River Egulya. The latter had the lowest critical load values. Therefore, it was identified as the most sensitive one to both Pb and Cd adverse effects.

Metal fluxes forming critical loads are relatively permanent in the long term because of the stable vegetation growth and outflow rate, and in that way, they do not change dramatically in the course of time. This was not the trend for the atmospheric depositions because they depend on the precipitation amount and heavy metal concentrations. Table 2 shows the results of the metal deposition rate and exceedance of the critical loads for the whole investigated period.

**Table 2.** Pb and Cd depositions and critical load exceedance for Pb and Cd for the period 2006–2008.

Ecosystem	Year	Dep(Pb) (g ha^−1^ yr^−1^)	Dep(Cd) (g ha^−1^ yr^−1^)	CL(Pb)ex (g ha^−1^ yr^−1^)	CL(Cd)ex (g ha^−1^ yr^−1^)
Petrohan dam	2006	31.32	2.24	7.49	1.22
	2007	9.31	1.03	−14.43	0.02
	2008	12.83	0.99	−10.92	−0.03
					
Ogosta dam	2006	30.22	1.31	7.49	0.50
	2007	12.97	0.79	−14.43	−0.03
	2008	3.18	0.53	−10.92	−0.21
					
The River Martinovska	2006	16.15	1.79	7.49	0.66
	2007	5.82	0.73	−14.43	−0.41
	2008	3.74	0.75	−10.92	−0.39
					
The River Egulya	2006	16.43	2.09	0.03	1.13
	2007	14.18	1,67	−2.19	0.89
	2008	13.37	0.74	−3.00	−0.04

The deposition rates for 2006 for both Pb and Cd (Table 2) were the highest, because of the highest precipitation values (except the River Egulya) and the highest metal concentrations. Exceedances were detected for the four ecosystems for the two metals (except the River Martinovska in respect of Pb). It was found that for 2007 and 2008, the metal concentrations (except Petrohan dam in respect of Pb) and the deposition rates decreased. Therefore, critical load exceedance was not detected, i.e. its value was negative. This implies that the metal input was lower than the critical loads, and there was no metal accumulation and risk of harm for the ecosystems.

As already pointed out, of the four investigated ecosystems, the most sensitive one was the River Egulya. Nevertheless, its critical loads exceedance was found to be the lowest. The highest exccedance (11.93 g ha^−1^ yr^−1^) was calculated for Ogosta dam, which was the second most-sensitive ecosystem. Its precipitation amount was the lowest but the Pb concentration was the highest, and in that way the metal input in the ecosystem was higher than the one allowing normal ecosystem functioning. The most tolerant ecosystem was Petrohan dam; however, for 2006 exceedance for Pb was determined (7.49 g ha^−1^ yr^−1^). The only ecosystem without exceedance was the River Martinovska. The reason for that was the lowest Pb precipitation concentration regardless of the high precipitation amount.

All calculated exceedances of critical loads for Pb for 2007 and 2008 were negative for all investigated ecosystems. A decreasing trend of metal deposition rates and an increase in negative values of exceedances were found. This could be considered a good indication for sustainable development and lack of risk of damage for the ecosystems. A stable trend of deposition rate was not observed only for the Petrohan dam ecosystem because of the changeable Pb concentrations and high precipitation amount. Nevertheless, this was the most tolerant ecosystem in respect of Pb, and the probability of metal accumulation is very small, taking into account the decreasing metal emission in the context of the convention on LRTAP.

In respect of Cd, for all investigated ecosystems there was exceedance of critical loads for 2006. Both the precipitation amount and the concentrations were highest in that year, and the metal deposition input was higher than that allowing normal functioning of the ecosystems. This indicates Cd net accumulation and potential risk for sensitive organisms. Even the River Martinovska ecosystem, which was the most tolerant, received higher metal deposition than its critical load value. For the most sensitive ecosystem the River Egulya, the exceedance of critical load was the highest (1.13 g ha^−1^ yr^−1^). A trend for decreasing Cd precipitation concentrations and amount as well as deposition rate was observed (except the River Egulya in 2007). In that way, the probability of Cd accumulation and adverse effect could decrease. Nevertheless, the results showed exceedance of the critical loads for Cd for two years for two of the four ecosystems: the River Egulya and Petrohan dam. The former was most sensitive but the latter received higher metal input because of the high precipitation amount. The lack of exceedance for 2008 was not so satisfying because of the very small difference between the deposition rate and the critical load value. Only for the Ogosta dam ecosystem there was nearly 30% lower metal input than the critical load.

An ecotoxicological test for water quality was carried out in 2011. In May, water samples from the Petrohan dam, Ogosta dam and the River Martinovska were investigated. None of the samples was inhibitory to the test micro-organism, based on optical density or direct diffusion method. The indirect Koch method for the quantity assessment of micro-organisms showed similar values of 10^8^ CFU cm^−3^ in all of the tested water bodies.

**Figure 2.**
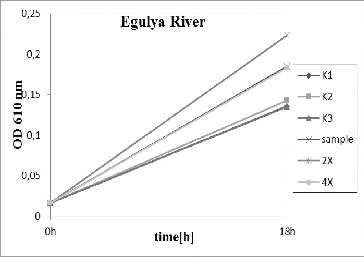
*Pseudomonas putida* growth inhibition tests for the River Egulya. K1, K2, K3 – triplicate tests for the control; sample – undiluted water; 2× – water diluted twofold; and 4× – water diluted fourfold.

**Figure 3.**
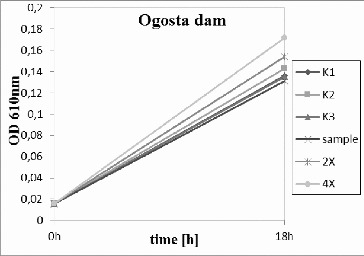
*Pseudomonas putida* growth inhibition tests for Ogosta dam. K1, K2, K3 – triplicate tests for the control; sample – undiluted water; 2× – water diluted twofold; and 4× – water diluted fourfold.

**Figure 4.**
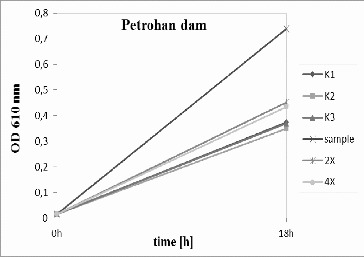
*Pseudomonas putida* growth inhibition tests for Petrohan dam. K1, K2, K3 – triplicate tests for the control; sample – undiluted water; 2× – water diluted twofold; and 4× – water diluted fourfold.

The River Egulya water (Figure 2) did not inhibit bacterial growth in comparison to the control variant. In contrast, diluted and undiluted samples showed higher density than the control. It could be suggested that some organic matter dissolved in natural water stimulated the growth of the tested bacteria *P. putida*, as the experimental medium is synthetic without growth factors. Similar results were obtained for Ogosta dam (Figure 3) and Petrohan dam (Figure 4).

**Figure 5.**
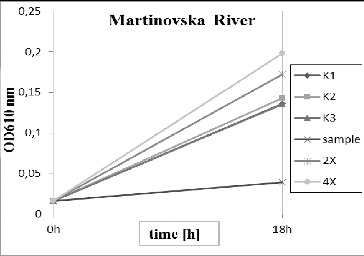
*Pseudomonas putida* growth inhibition tests for the River Martinovska. K1, K2, K3 – triplicate tests for the control; sample – undiluted water; 2× – water diluted twofold; 4× – water diluted fourfold.

At the beginning of September, the water samples were investigated again because of a long period of dry and warm weather and low precipitation amount. The samples from Ogosta dam and the River Egulya were similar: there was stimulation of the test organism growth instead of inhibition. As the test was conducted at the time of low water quantity, it could be inferred that there was no toxic effect on the microbial populations in the investigated ecosystems. As prokaryotic micro-organisms are very adaptable to environmental conditions, it is not possible to extrapolate these results onto the higher organisms in these aquatic ecosystems. The high temperature and suspended particles could be toxic to crustaceans and fishes. As such tests were not conducted, a conclusion that the waters were nontoxic cannot be made.

In contrast, the water from the River Martinovska (Figure 5) was toxic for the tested bacteria. The optical density of bacterial suspension was almost unchanged on the 16th h in the sample with natural water; the twofold and fourfold diluted samples did not show inhibition effects. The results from the turbidimetric measurement were also proved by the indirect method of Koch. On the agar plates with rich nutrient medium, the Martinovska sample had only 20% of the CFUs per cm^3^ in comparison with the quantity (CFU cm^−3^) in the control variant.[5] It can be concluded that, during low water, the water of the River Martinovska was toxic for the microbial community. Additional evidence was provided by the disk-diffusion method. A sterile zone was formed from undissolved water on the solid nutrient medium seeded with the tested organism. In this case, the test with *Daphnia magna* and *Danio rerio* is recommendable to determine the effects on higher organisms.[10] As could be reviewed by Bondarenco et al. [11], these two tests are the most widely used ones in toxicity determination all over the world.

## Conclusions

All of the four studied ecosystems were observed to be more sensitive to cadmium than to lead deposition. Concerning lead (Pb), risk for harmful effects was not found because its depositions were below the critical load values. Cd adverse effects on aquatic organisms with regard to critical load exceedance are highly probable, and special attention should be paid to reducing its emission. The *Pseudomonas putida* growth inhibition test detected a lack of toxicity for all studied ecosystems with the exception of the low water September sample of the River Martinovska. The results obtained by microbial test should be confirmed by other sensitive tests using other bioindicator organisms like *Dafnia magna* and *Danio rerio* to determine the extent of necessary dilution of water. The results obtained could enable national water control authorities to identify the water bodies at risk, consistently subjected to heavy metal deposition in view of pollution abatement policy.
